# Pharmacological targeting transient receptor potential canonical channel 6 modulates biological behaviors for cervical cancer HeLa and SiHA cell

**DOI:** 10.1186/s12935-022-02556-4

**Published:** 2022-04-07

**Authors:** Li-ping Bai, Ya-li Chen, Ai Zheng

**Affiliations:** Department of Gynaecology and Obstetrics, West China Second University Hospital, Sichuan University, Key Laboratory of Birth Defects and Related Diseases of Women and Children (Sichuan University), Ministry of Education, No. 20, Renmin South Road, Wuhou District, Chengdu, 610041 China

**Keywords:** Cervical cancer, TRPC6, Ca2 + channel, SKF-96365, OAG, Proliferation, Migration

## Abstract

**Background:**

This study aimed to observe the effect of transient receptor potential canonical channel 6 (TRPC6) antagonist 1-(β-[3-(4-method-phenyl) propoxy]-4-methoxyphenethyl)-1H-imidazole hydrate (SKF-96365) and its agonist 1-oleoyl-2-acetyl-sn-glycerol (OAG) on the proliferation of cervical cancer cell lines HeLa and SiHa, deoxyribonucleic acid (DNA) synthesis, cell migration, and TRPC6 expression.

**Method:**

Real-time quantitative polymerase chain reaction (RT-qPCR) and western blotting were used to detect the expression of TRPC6 in HeLa and SiHa cells. The tetrazolium salt 3-(4,5-dimethylthiazol-2-yl)-2,5-diphenyltetrazolium bromide (MTT) assay, the 5-ethynyl -2'- deoxyuridine (EdU) fluorescence detection assay, and a scratch test were used to detect the changes of proliferation, DNA synthesis and cell migration of HeLa and SiHa cells after SKF 96,365 and OAG acted on HeLa and SiHa cells for different lengths of time. RT-qPCR was used to detect expression changes of TRPC6 SKF-96365 and OAG treated HeLa and SiHa cells.

**Results:**

TRPC6 was expressed both in HeLa and SiHa cells. The MTT assay showed that after 24 h of SKF-96365 treatment, compared with the control group, the proliferation of HeLa and SiHa cells was inhibited, and there was a statistically significant difference (p < 0.05). After 24 h of OAG, compared with the control group, the proliferation of HeLa and SiHa cells had increased, and there was a statistically significant difference (p < 0.05). EdU fluorescence detection showed that SKF-96365 could inhibit the DNA synthesis of HeLa and SiHa cells, and OAG could promote the DNA synthesis of HeLa and SiHa cells (p < 0.05) in HeLa and SiHa cell lines.

**Conclusion:**

The high expression of calcium channel TRPC6 in HeLa and SiHa tissues may be related to the malignant behavior of cervical cancer cell lines HeLa and SiHa. This calcium channel may be a new target for the prevention and treatment of cervical cancer.

## Background

Cervical cancer is the most common gynecological malignant tumor. It has the second highest incidence rate of female malignant tumors, only secondary to breast cancer. In cells, calcium is closely associated with tumors, and it is directly involved in the regulation of tumor growth, invasion, metastasis, and differentiation [1–5]. Transient receptor potential (TRP) channels are considered to be the most likely store-operated channels and the receptor-operated channels whose molecular basis is expressed in a variety of cells [6, 7]. In recent years, studies have found that the over-expression of TRPC6 promotes tumor proliferation, growth, and invasion [8–21]. The expression of TRPC6 in cervical cancer tissues is greater than in normal cervical tissues [20, 21], and verapamil, a calcium channel antagonist, has been found to reduce the invasiveness of HeLa cells by co-culture in HeLa cells. To further explore the role of TRPC6 channel in the proliferation, growth, and invasion of cervical cancer, this study used TRPC6 channel inhibitor 1-(β-[3-(4-method-phenyl) propoxy]-4-methoxyphenethyl)-1H-imidazole hydrate (SKF-96365) and its agonist 1-oleoyl-2-acetyl-sn-glycerol (OAG) to co-culture with the cervical cancer cell lines HeLa and SiHa and observed their effects on the proliferation and migration ability of these two cell lines.

## Materials and methods

### Preparation of common reagents

#### Preparation of complete medium containing SKF-96365 20 μmol/L

Based on its molecular weight, 5 × 10^−3^ g of SKF-96365 (Sigma-Aldrich, the USA) was dissolved in 3,100 μL dimethyl sulfoxide (DMSO), at 4 mmol/L (200 working concentration) and aliquoted in 10 autoclaved Eppendorf (EP) tubes. It was stored at − 20 °C and protected from exposure to light for later use.

Ninety mL of Roswell Park Memorial Institute (RPMI) 1640 basal medium, stored a − 20 °C, was thawed at 4 °C, and 10 mL (1 mg/ml) of calf serum (Minhai Biological Technology Co., Ltd., China) and SKF-96365 250 ul (final concentration: 20 μmol/L) dissolved in DMSO at 200 times concentration, were added to it.

#### Preparation of complete medium containing OAG 50 μmol/L

Based on its molecular weight, 10 × 10^−3^ g of OAG (Sigma-Aldrich, the USA) was dissolved in 2,500 μL DMSO at 10 mmol/L (200 working concentration) and aliquoted in 10 autoclaved EP tubes. It was stored at − 20 °C and protected from exposure to light for later use.

Ninety mL of RPMI 1640 basal medium stored at − 20 °C was thawed at 4 °C, and 10 mL (1 mg/ml) of calf serum and 500 ul (final concentration 50 μmol/L) of OAG in 200-fold DMSO were added to it.

### Cell culture

HeLa and SiHa cells (human cervical cancer HeLa and SiHa cell lines stored in the Cardiovascular Laboratory II, West China Hospital, Sichuan University) were routinely cultured in RPMI 1640 medium(GIbco, Inc,USA) containing 10% inactivated newborn calf serum(Minhai Biological Engineering Co., LTD, China). The cells were incubated in a sterile incubator at 37 °C and 5% CO_2_. After 80–90% of cell adhesion, 0.25% trypsin (Sigma-Aldrich, the USA) was used for digestion and passage for subsequent tests.

### The real-time polymerase chain reaction detection of the expression of TRPC6 in HeLa and SiHa cells

#### Using TRIzol Reagent to extract total ribonucleic acid from cells

Total ribonucleic acid (RNA) was extracted from HeLa and SiHa cells in accordance with the operation instructions of TRIzol® reagent (Invitrogen, the USA).

#### The reverse transcription of RNA into complementary deoxyribonucleic acid

RNA was taken out from the − 70 °C refrigerator and thawed on ice. The volume corresponding to 1.5 μg RNA in the reverse transcription reaction system was calculated according to the RNA concentration determined by ultraviolet spectrophotometry, and the total reaction system mixture was finally 20 µL. See Table 1 for the specific reaction system.

**Table 1:** RT reaction system

Reagent	Volume(µL)
5 × RT buffer	4.0
dNTP mixture(l0mM)	2
RT-enhancer	0.5
Super RI	0.5
Oligo(dT)18	1.0
Rever Tra Ace \、/*/VC 外	1.0
1.5 µg RNA Sample	X
RNAase free ddH_2_O	(ll–X)
Total volume	20 µL

After being shaken up and mixed for 5 s, the reverse transcribed complementary deoxyribonucleic acid mixture was kept at 42 °C for 50 min and then stored in the refrigerator at − 20 °C.

#### Real-time polymerase chain reaction

The primer sequence was obtained through the Pubmed gene pool, and the accuracy was verified by the Basic Local Alignment Search Tool before it was sent to Invitrogen (Shanghai) for synthesis. The specific primers are shown in Table 2. The total volume of real-time PCR was 20 μL, and the concentration of diluted primer was 10 pmol/μL. The real-time polymerase chain reaction (RT-PCR) reaction system can be seen in Table 3.

**Table 2:** PCR primers

Gene	Primer sequence (5'-3')	Product size(bp)
TRPC6	F:GCCAATGAGCATCTGGAAAT	152
	R:AACCTCTTGCCTTCAGCAAA	
β-actin	F:ACTATCGGCAATGAGCGGTTC	77
	R:ATGCCACAGGATTCCATACCC

**Table 3:** RT-PCR reaction system

Component	Volume (pL)	Final Concentration
2.5 × Real Master Mix 20 × SYBR solution	10	L ×
Primer#l (10 pmol/µL)	0.5	0.25 µM
Primer#2 (10 pmol/µL)	0.5	0.25 µM
|cDNA	1	
ddHzO	8	
Total volume	20	

Semi-quantitative PCR amplification was performed on a quantitative PCR instrument (BIO-RAD). The temperature gradient was verified to find the optimal annealing temperature. The amplification condition was pre-denaturation at 95 °C for 2 min and then entered the cycle of 95 °Cfor 10 s, 57 °C for 10 s, and 72 °C for 15 s, for a total of 40 cycles.

#### PCR product identification

First, 0.375 g agarose was added to 15 mL TAE buffer, which is a mixture of Tris base, acetic acid, and ethylenediaminetetraacetic acid, together with a small amount of pure water, and the solution was heated to boiling point, then cooled in an electrophoresis plate; when it had cooled, 0.5 μL 6 × loading buffer, 2.5 μL RT-PCR product, and 4 μL of TAE buffer were added to the jack, and electrophoresis was performed at 90 v for about 25 min. The PCR product was then immersed in ethidium bromide solution (Amresco LLC, the USA) for 30 min, and then imaged using a Bio-Rad gel electrophoresis imager. According to the size of the primer, it was judged whether the PCR product was consistent with the electrophoresis band.

#### RT-PCR data processing

The gene level was expressed as an RT-PCR result (2ΔΔ Ct value), and the ratio of the control group to the internal reference gene β-actin was 1. The relative expression level of TRPC6 in each group was calculated. The result was the ratio relative to the expression level of the corrected sample, and the formula used was as follows:

$${\text{Ratio = }}\frac{{(2)\;\Delta {\text{ct}}\,{\mkern 1mu} {\text{target}}\,{\mkern 1mu} {\text{(control - expt)}}}}{{(2)\;\Delta {\text{ct}}{\mkern 1mu} \,{\text{reference}}{\mkern 1mu} \,{\text{(control - expt)}}}}$$

where target was the target gene TRPC6, reference was the internal reference gene β-actin, and control and expt are the Ct values of the control group (the ordinary medium group) and the experimental group (with the addition of SKF-96365 or OAG), respectively. The resulting ratio was the expression of the target gene in the test sample relative to the calibration sample. In this experiment, the result was the ratio of the relative expression levels of the target gene TRPC6 and the control group.

### The determination of TRPC6 expression in HeLa and SiHa cells using western blotting

After the HeLa and SiHa cells were cultured to the logarithmic phase, protein quantification was performed by using extracted proteins, followed by sodium dodecyl sulphate–polyacrylamide gel electrophoresis and transmembrane. Western hybridization, electrochemiluminescence imaging (Pierce, the USA), and fixation were performed, using rabbit anti-TRPC6 monoclonal antibody (Epitomics, the USA), β-actin antibody, and mouse anti-rabbit immunoglobulin G antibody (Santa Cruz, the USA), to determine the TRPC6 expression in the HeLa and SiHa cells.

### The detection of the proliferation of HeLa and SiHa cells

The HeLa and SiHa cells in the logarithmic phase were counted after trypsin digestion and then inoculated into 96-well plates for culture at a rate of 5 × 103 cells/well. After 70–80% of the cells had adhered to the wall, RPMI 1640 medium free of calf serum was added to synchronously culture the cell cycles for 11 h. After the cells were washed twice with phosphate buffered saline (PBS), SKF-96365 or OAG medium were added to the 96-well plates, and RPMI 1640 medium containing the same concentration of DMSO was used as the control group. After 24 h, the culture medium was discarded and the cells were washed twice with PBs, after which 20 μL of tetrazolium salt 3-(4,5-dimethylthiazol-2-yl)-2,5-diphenyltetrazolium bromide (MTT) (Sigma-Aldrich, the USA) (5 mg/mL) was added to each well, and the incubation was continued. After 4 h, 150 μL DMSO was added to each well, which was shaken for 10 min to dissolve the crystals. The 96-well plate was placed on a full-automatic microplate reader to determine the absorbance (A) value of each well. The colorimetric wavelength of 570 nm was selected, and the blank control was adjusted to zero. The optical density value of each well was measured. Four complex holes were set up, and the tumor cell proliferation inhibition rate was calculated

$${\text{Tumor cell proliferation rate }} = \left( {{\text{control group A57}}0 \, - {\text{ blank well A57}}0} \right) \, - \, \left( {{\text{experimental group A57}}0 \, - {\text{ blank well A57}}0 \, } \right) \times {1}00\%$$

(Control group A570 − blank well A570).

### The detection of the proportion of HeLa and SiHa cells in the S phase

Cell culture: HeLa and SiHa cells in the logarithmic phase were counted after trypsin digestion and then inoculated into 96-well plates for culture at a rate of 5 × 103 cells/well. After 70–80% of the cells adhered to the wall, RPMI 1640 medium free of calf serum was added to synchronously culture the cell cycles for 11 h. SKF-96365 or OAG medium was added to 96-well plates with RPMI 1640 medium containing the same concentration of DMSO as a control. The cells were incubated for 24 h. The 5-ethynyl -2'- deoxyuridine (EdU) solvent (reagent A) (Guangzhou RiboBio Co., Ltd., China) was diluted with 1640 basal medium without double antibodies at a ratio of 1000:1, and an appropriate amount of 50 μmol/L EdU medium was prepared. Next, 100 μL of 50 μmol/L EdU medium was added to each well, and the cells were incubated for 2 h, after which the medium was discarded. The cells were washed twice with PBS, for 5 min each time. Fifty μL cell fixative (40 g/L paraformaldehyde) was added to each well, and they were incubated at room temperature for 30 min, then the fixative was discarded. Each well was incubated with 50 μL glycine (2 mg/mL) in a shaker for 10 min, and then the glycine solution was discarded. One hundred μL PBS was added to each well, and they were washed using an orbital shaker for 5 min, and then the PBS was discarded. This was repeated once. Then each well was incubated with 100 μL osmotic agent (PBS containing 0.5% TritonX-100) in an orbital shaker for 10 min, and then washed once with PBS for 5 min. Each well had 100 μL Apollo staining reaction solution added to it, after which they were incubated in the dark at room temperature in a shaker for 30 min, and then the staining reaction solution was discarded. Each well was washed 2–3 times with 100 μL of osmotic agent (PBS containing 0.5% TritonX-100) in an orbital shaker, for 10 min each time. Reagent F was diluted with ddH2O in a ratio of 100:1, and an appropriate amount of 1 × Hoechst 33342 reaction solution was prepared and protected from exposure to light. Each well had 100 μL 1 × Hoechst 33342 reaction solution added to it, and the cells were then incubated in the dark at room temperature in a shaker for 30 min, after which they were washed twice with PBS. Finally, the solution was observed and photographed under a Nikon fluorescent inverted microscope, and Image-Pro Plus 6.0 professional image analysis software (IPP) was used to calculate the number of positive cells.

### The detection of HeLa and SiHa cell migration using a scratch damage test

HeLa and SiHa cells in the logarithmic phase were counted after trypsin digestion and then inoculated into 6-well plates for culture at a rate of 5 × 105 cells/well. After 70–80% of the cells adhered to the wall, RPMI 1640 medium free of calf serum was added to synchronously culture the cell cycles for 11 h. Then a 20 μL sterile pipettor head was used to make an "1" scratch, and the exfoliated cells were rinsed with PBS. The experimental group was treated with 2 mL RPMI 1640 complete medium containing different drugs, and the control group was treated with 2 mL RPMI 1640 complete medium containing the same volume of DMSO. Each group had 10 mM hydroxyurea (Sigma-Aldrich, the USA) added to it. The markers were photographed under a Nikon fluorescent inverted microscope and incubated in a sterile incubator at 37 °C and 5% CO2. After 24, 48, and 72 h, more photos were taken in the marked visual field. The migration distance was measured using IPP software.

### Statistical analysis

All the experiments were repeated at least three times, and the results were expressed as ± S. All the parameters were analyzed using the SPSS 17.0 one-way analysis of variance statistical software package. A value of p < 0.05 indicated that the results were statistically significant.

## Results

### RT-PCR: the expression of TRPC6 in HeLa and SiHa cells

**Fig. 1:**
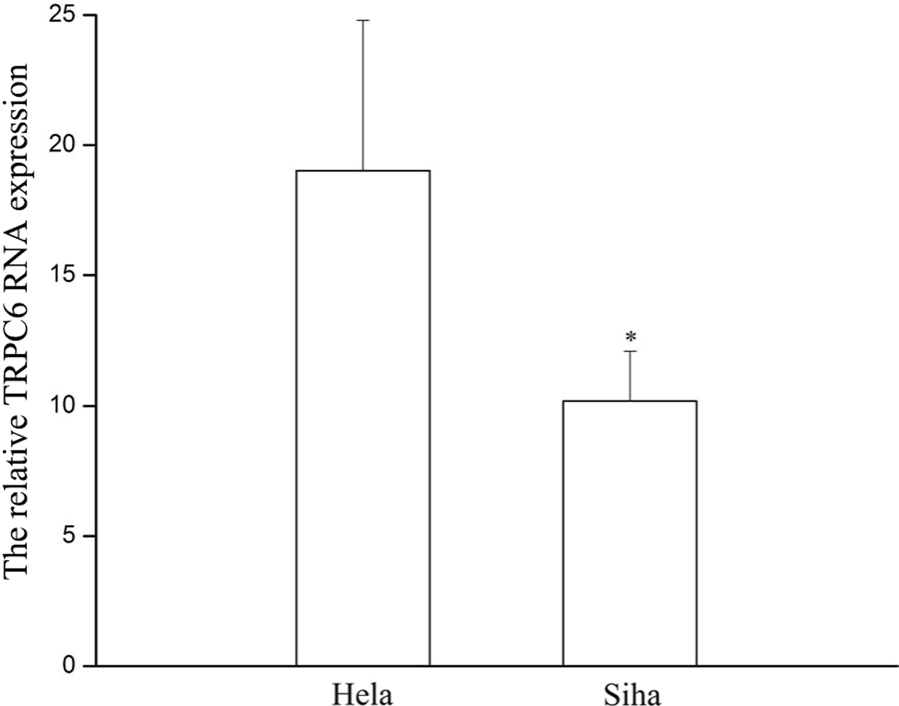
The expression of TRPC6 RNA in HeLa and SiHa cells. *p < 0.05 vs HeLa, n = 6

HeLa and SiHa cells were cultured to the logarithmic phase, and the expression of TRPC6 in the cells was detected using RT-PCR. The baseline Cycle threshold (Ct) value in Hela cells was about 23.00 and the baseline CT value in Siha cells was about 24.42. The results shown in Fig. 1 are the average relative values of expression after correction. The expression of TRPC6 was detected at the gene level both in HeLa and SiHa cells.

### Western blotting: the expression of TRPC6 in HeLa and SiHa cell lines

**Fig. 2:**
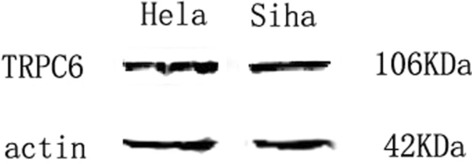
The expression of TRPC6 protein in HeLa and SiHa cells

The expression of TRPC6 protein in HeLa cells was detected by western blotting, and the results confirmed that TRPC6 protein was expressed both in HeLa and SiHa cells (Fig. 2).

### MTT: the effects of SKF-96365 and OAG on the proliferation of HeLa and SiHa cells

After SKF-96365 (final concentration 20 μmol/L) was added and then left for 24 h, compared with the control groups, the proliferation of both HeLa and SiHa cells was inhibited, with the differences being statistically significant in both cases (p < 0.05). After 24 h of OAG treatment (final concentration 50 μmol/L), compared with the control groups, the proliferation of HeLa and SiHa cells increased, with the differences being statistically significant in both cases (p < 0.05). That is to say, in HeLa and SiHa cell lines, 20 μmol/L SKF-96365 was able to inhibit the proliferation of HeLa and SiHa cells, and 50 μmol/L OAG was able to promote cell proliferation. The results are shown in Table 4.

**Table 4:** Effects of SKF96365 and OAG on HeLa and Siha cell proliferation

	Group	n	A570	Inhibition Rate(%)
Hela	SKF96365 Control group	4	0.7630 ± 0.0523	0
	SKF96365 experimental group	4	0.6149 ± 0.0269*	20.55 ± 3.30*
	OAG Control group	4	0.5982 ± 0.0302	0
	OAG Experimental group	4	0.7366 ± 0.0304*	− 25.19 ± 1.64*
Siha	SKF96365 Control group	4	1.2576 ± 0.1910	0
	SKF96365 Experimental group	4	0.9284 ± 0.0289*	25.65 ± 11.39*
	OAG Control group	4	1.1938 ± 0.0222	0
	OAG Experimental group	4	1.3825 ± 0.1152*	− 16.20 ± 7.78*

*P < 0.05 vs Control

SKF-96365 = 1-(β-[3-(4-method-phenyl) propoxy]—4—methoxyphenethyl)—1H—imidazole hydrate

OAG = 1-oleoyl-2-acetyl-sn-glycerol

### EdU: the effects of SKF-96365 and OAG on DNA synthesis in HeLa and SiHa cells

**Fig. 3:**
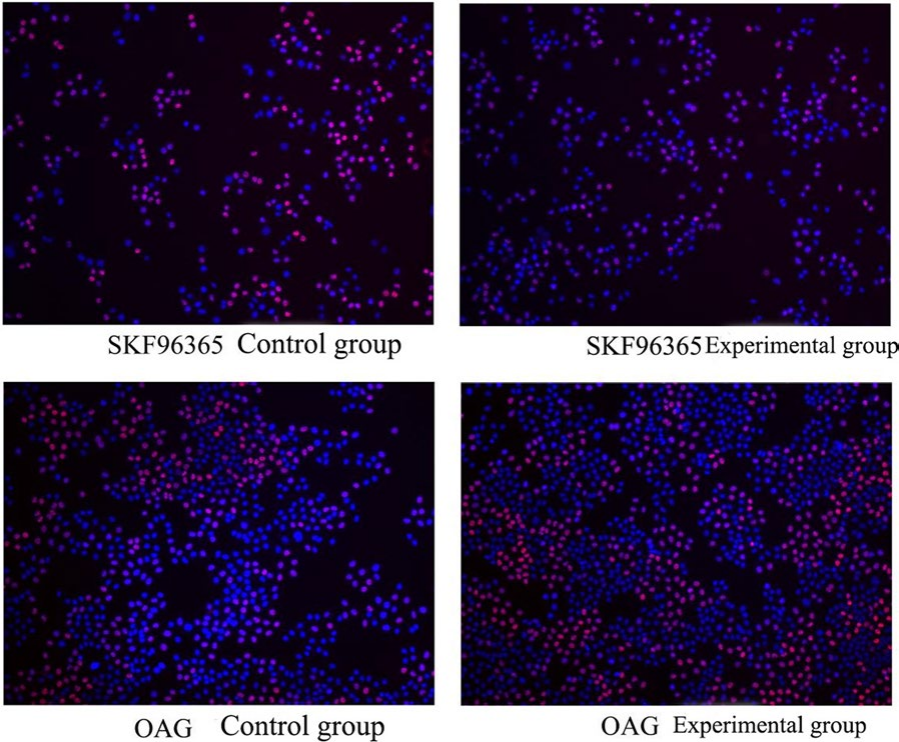
HeLa cells in S phase after SKF-96365, OAG treated for 24 h 100 ×

**Fig. 4:**
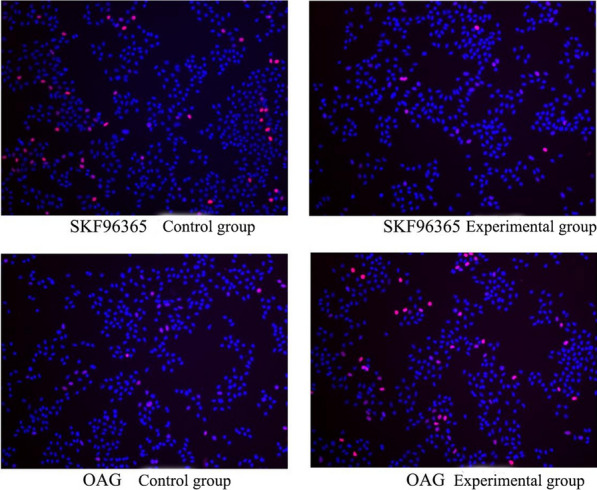
SiHa cells in S phase after SKF-96365, OAG treated for 24 h 100 ×

After the HeLa and SiHa cells were treated with SKF-96365 and OAG for 24 h, the cells were counted using IPP software to obtain the proportion of cells in the S phase. After treatment with SKF-96365, the proportion of HeLa cells in the S phase was 69.8 ± 5.0%, which was lower than the proportion in the control group (80.7% ± 9.1%) (p < 0.05). After treatment with SKF-96365, the proportion of SiHa cells in the S phase was 9.2% ± 0.9%, which was lower than the proportion in the control group (11.8% ± 1.8%) (p < 0.05). After treatment with OAG, the proportion of HeLa cells in the S phase was 70.1 ± 5.6%, which was higher than the proportion in the control group (57.4 ± 7.6%) (p < 0.05). After treatment with OAG, the proportion of SiHa cells in the S phase was 13.2% ± 2.3%, which was higher than the proportion in the control group (9.5% ± 1.5%) (p < 0.05). In other words, in HeLa and SiHa cell lines, 20 μmol/L SKF-96365 was able to inhibit the DNA synthesis of HeLa and SiHa cells, and 50 μmol/L OAG was able to promote cell DNA synthesis, as can be seen in Fig. 3 and 4.

### Scratch test: the effects of SKF-96365 and OAG on the migration of HeLa and SiHa cells

**Fig. 5:**
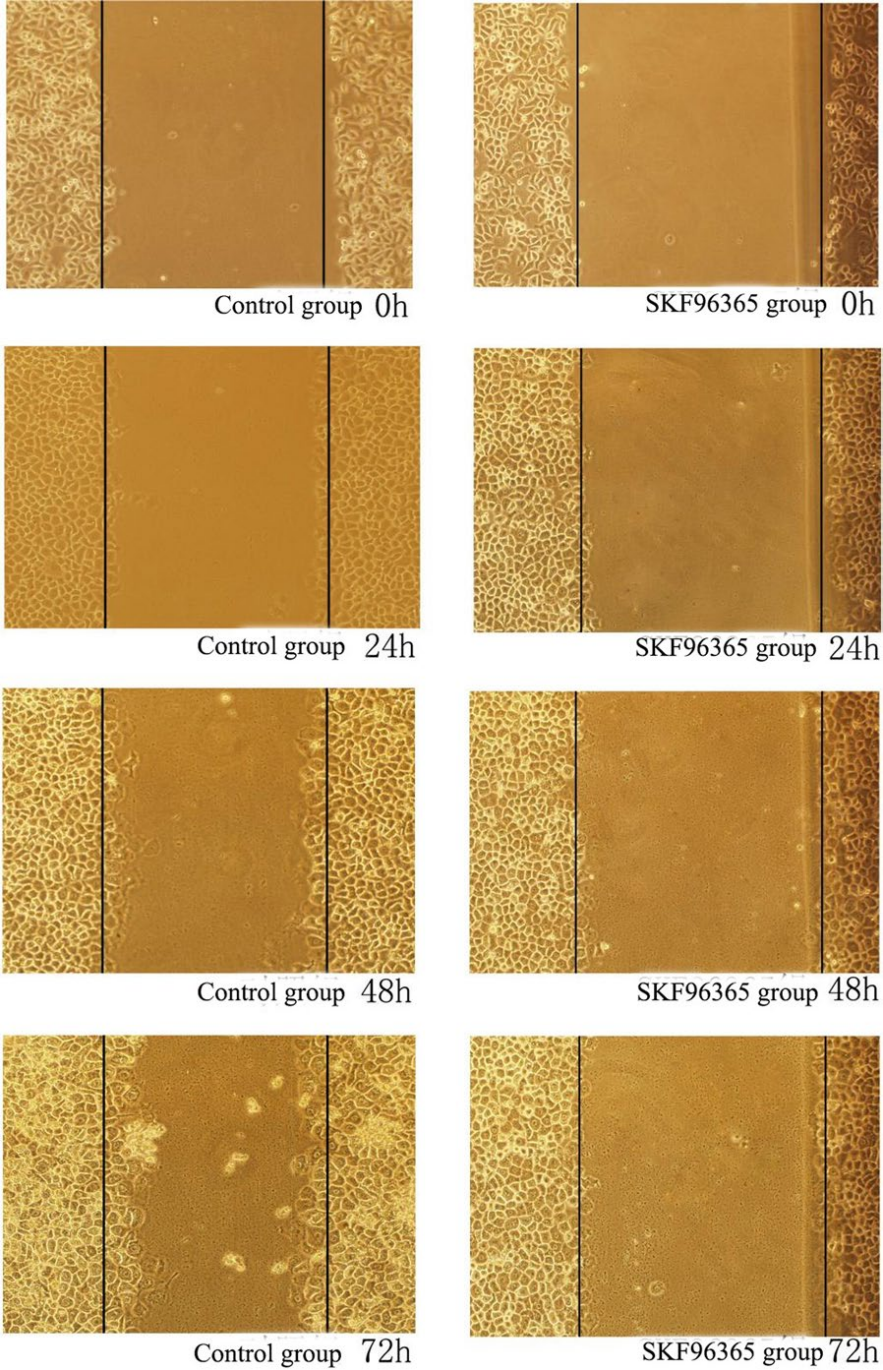
The effects of SKF-96365 on HeLa cell migration × 100

**Fig. 6:**
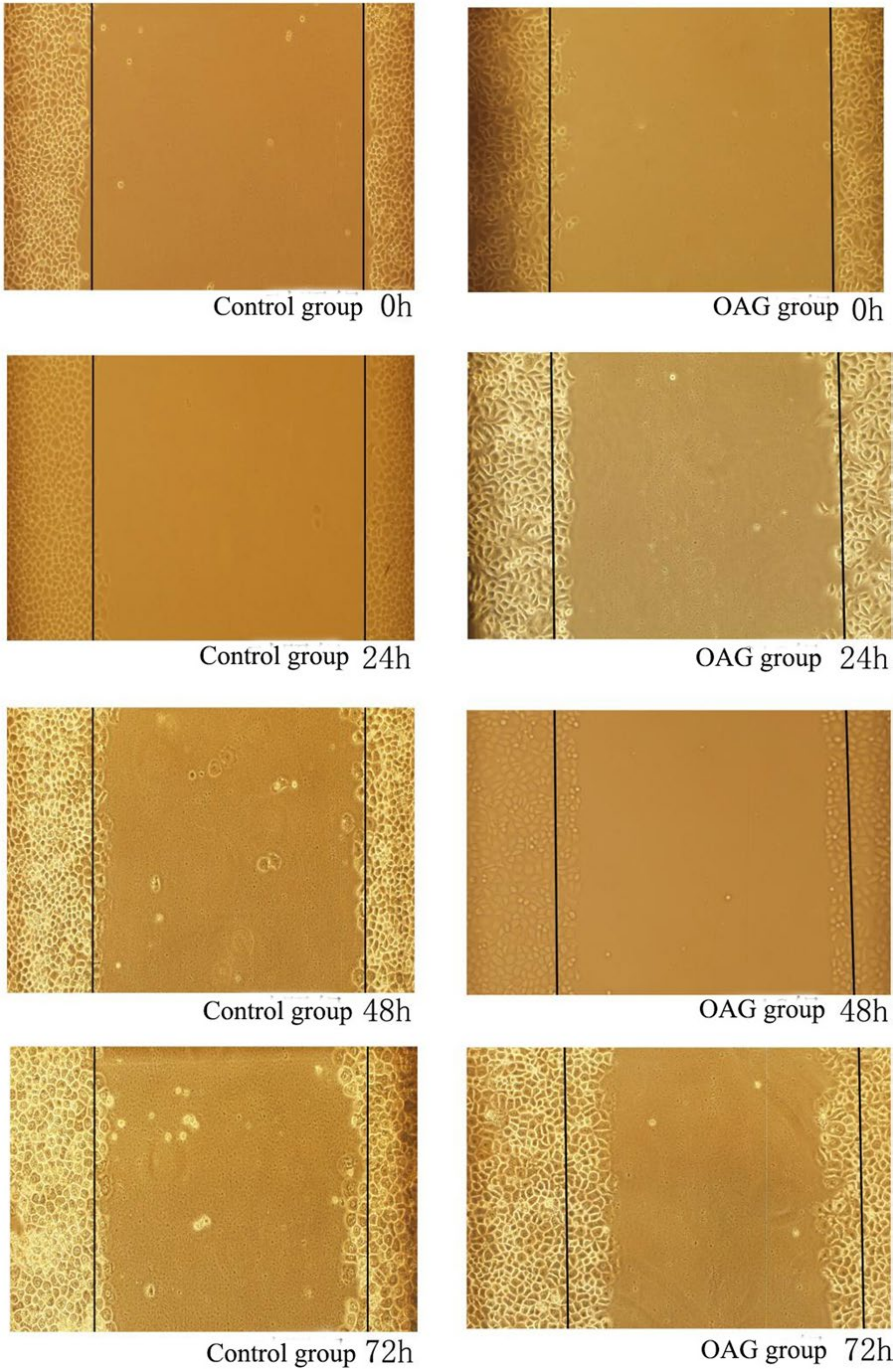
The effects of OAG on HeLa cell migration × 100

**Fig. 7:**
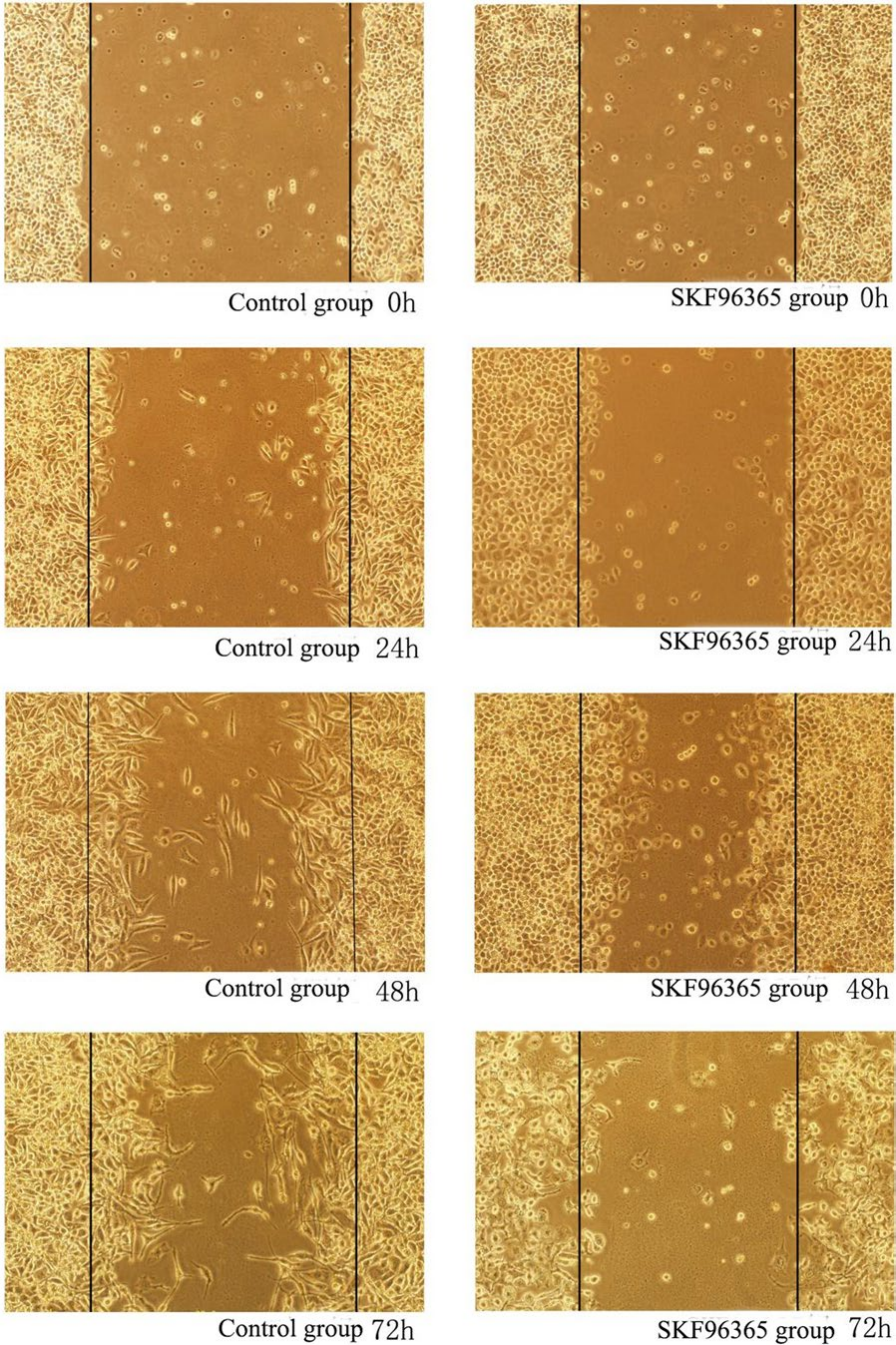
The effects of SKF-96365 on SiHa cell migration × 100

**Fig. 8:**
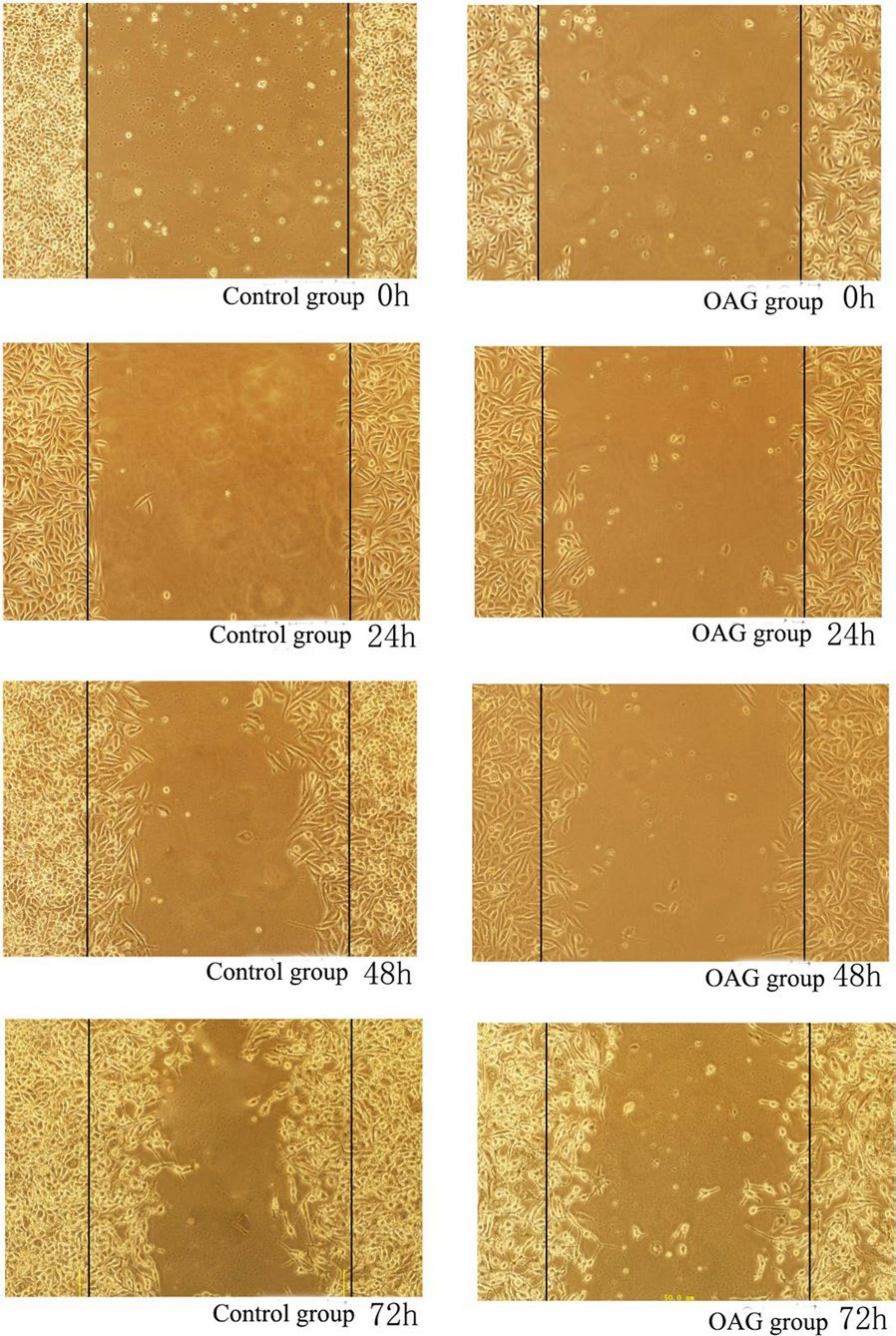
The effects of OAG on SiHa cell migration × 100

**Fig. 9:**
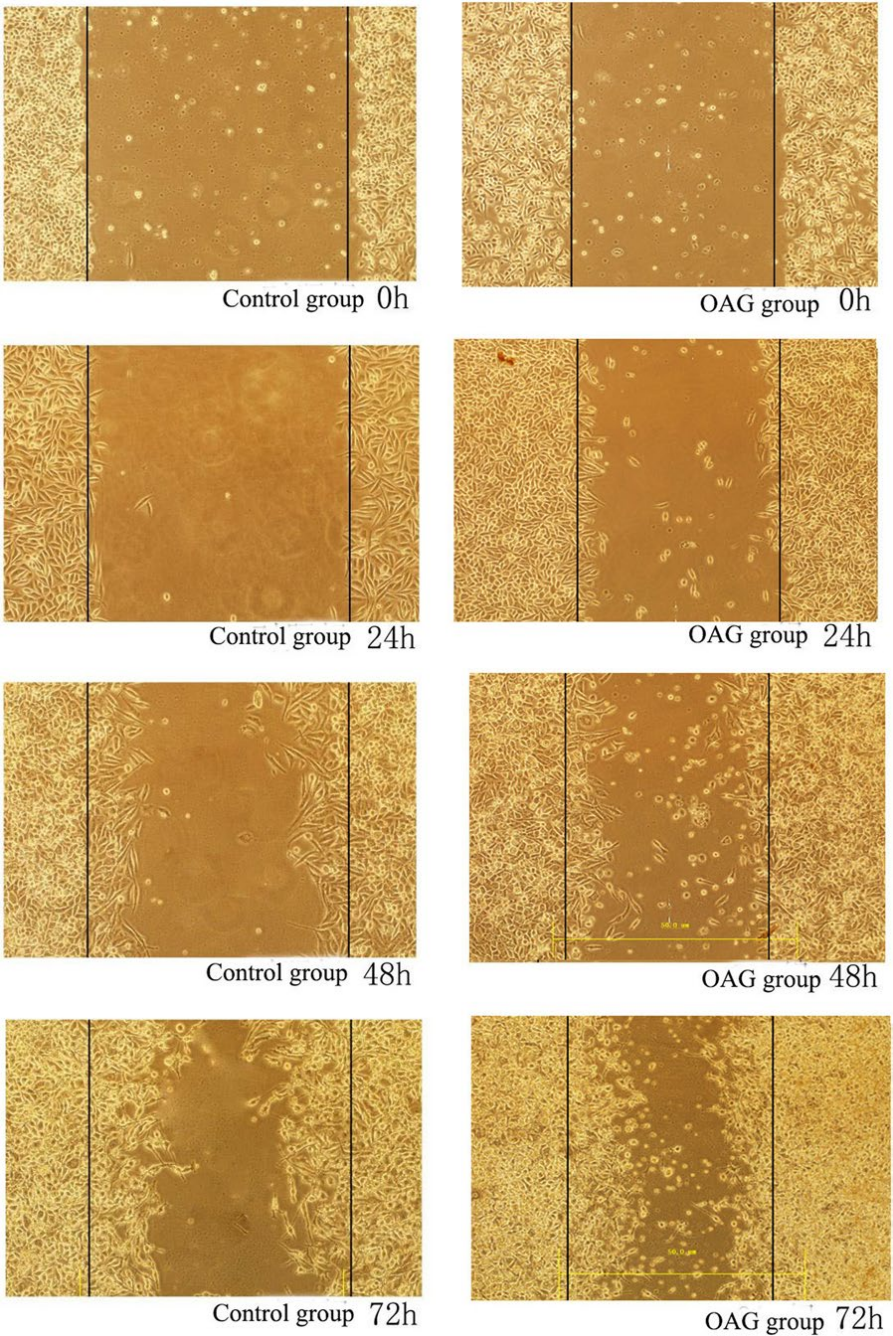
The effects of OAG (100 μmol/L) on SiHa cell migration × 100

After treating HeLa and SiHa cells with SKF-96365 and OAG for 24 h, 48 h and 72 h, compared with 0 h, the cells migrated to the scratch due to the natural healing movement of the cells. In HeLa and SiHa cell lines, 20 μmol/L SKF-96365 was able to inhibit the migration of HeLa and SiHa cells. OAG at 50 μmol/L promoted the migration of HeLa cells, but had no significant effect on the migration of SiHa cells. The results are shown in Table 5 and Figs. 5, 6, 7,8 and 9.

**Table 5:** Migration distance of Hela and Siha cells treated with SKF96365 and OAGmigration distance

Cells	Group	Migration distance(μm)		
		24 h	48 h	72 h
Hela	SKF96365 Control group	3.58 ± 0.73	5.54 ± 0.85	7.75 ± 1.47
	SKF96365 Experimental group	1.42 ± 0.19*	2.20 ± 0.52*	2.79 ± 0.26*
	OAG Control group	2.17 ± 0.51	3.46 ± 0.62	4.83 ± 1.34
	OAG Experimental group	4.83 ± 1.34*	6.29 ± 0.71*	12.04 ± 1.51*
Siha	SKF96365 Control group	6.67 ± 1.12	10.58 ± 1.61	14.72 ± 1.22
	SKF96365 Experimental group	3.88 ± 0.66*	7.04 ± 0.52*	5.54 ± 0.76*
	OAG Control group	2.46 ± 0.44	9.25 ± 0.57	11.71 ± 0.63
	OAG Experimental group (50 μmol/L)	6.67 ± 0.81*	9.83 ± 0.92	12.33 ± 0.71
	OAG Experimental group (100 μmol/L)	6.29 ± 0.71*	12.04 ± 1.5*^#^	15.29 ± 3.67

*P < 0.05 vs Control; ^#^*P* < 0.05 vs OAG group (50 μM)

## Discussion

TRPC6 is a nonselective cation channel located on the cell membrane. TRPC6 consists of six transmembrane helices and contains 931 amino acids [22, 23]. TRPC6 channels can be directly activated by diacylglycerol (DAG) and DAG analogue-OAG [24], and they can be inhibited by SKF-96365 [25].

In recent years, a number of studies have found that the expression of TRPC6 is increased in a variety of tumors and is related to malignant behavior. Shi et al. [9] found that the expression of the TRPC6 protein and messenger RNA (mRNA) in esophageal cancer tissue was significantly higher than that in normal esophageal tissue. After Shi et al. blocked the TRPC6 channel with SKF-96365, the peak Ca2 + concentration and Cdc2 kinase level in esophageal cancer cell lines Eca109 and TE1 were inhibited. In addition, the cells were blocked in the G2/M phase, and the cell growth cycle was inhibited [9, 10]. With the decrease of TRPC6 expression, the number of cells decreased significantly. At the same time, flow cytometry showed that most of the transfected TE1 cells were blocked at the G2/M phase. Moreover, the TRPC6 channel was blocked when esophageal squamous cell carcinoma cells were injected into nude mice, and the tumorigenicity was reduced [9]. Sun Yat-sen University Cancer Center studied 172 cases of freshly frozen specimens after resection of esophageal cancer [11], and it was found that the expression of TRPC6 mRNA and protein was increased in esophageal cancer tissues compared with para-carcinoma tissues. Moreover, the expression level of TRPC6 mRNA was correlated with the pathological stage and disease specific survival (DSS) of the patients. The 5-year DSS of patients with high TRPC6 mRNA expression was 42.1%, lower than that of patients with low TRPC6 expression (62.7%). Cox multivariate analysis showed that a high TRPC6 mRNA level was an independent risk factor for the prognosis of esophageal cancer. The expression of TRPC6 is also found to be higher in gastric, ovarian, prostate, liver, and breast cancers than it is in normal tissues. It is also related to the malignant behavior of tumors and mainly affects the behavior of tumor cells by mediating a change in Ca2 + flow [8–21].

The specific mechanism of TRPC6 in promoting the occurrence and development of tumors needs to be further studied. To date, studies have reported that the expression of TRPC6 channel in tumors is related to tumor-related factors, such as endothelial growth factor (EGF), vascular EGF, and platelet-derived growth factor [15, 23, 26–28]. In addition, the p53 gene directly binds to the reaction element of the TRPC6 promoter, which increases the expression levels of TRPC6 mRNA and protein and is related to the increase of intracellular calcium ion levels [29].

The previous study results of our group showed that TRPC6 was significantly higher in cervical cancer tissue than normal cervical tissue [20]. The TRPC6 gene and its protein were also expressed in cervical cancer HeLa cells. Immunohistochemical analysis of paraffin sections showed that the high expression of TRPC6 had a significant correlation with lymphovascular space invasion in cervical cancer (p < 0.05), but it had no significant correlation with the International Federation of Obstetrics and Gynecology stage, the age of the patient, and pelvic lymph node metastasis (p > 0.05). After HeLa cells were treated with the non-selective calcium channel antagonist verapamil, the proliferation and migration of the cells were blocked [21].

This study found that TRPC6 was expressed in both HeLa and SiHa cells. The TRPC6 channel is inhibited by SKF-96365, which can inhibit the proliferation, DNA synthesis, and migration of HeLa and SiHa cells. Activation of the TRPC6 channel by OAG promoted the proliferation and DNA synthesis of HeLa and SiHa cells. It also promoted the migration of HeLa cells, but its effect in promoting cell migration in SiHa cells was not significant.

## Conclusion

In conclusion, the high expression of the TRPC6 channel in HeLa and SiHa cells may be related to its malignant behaviors. As a component of cation channel complex, the action mechanism of TRPC6 is still unclear. Recent studies have found that its physiological effect is closely related to the mediated calcium ion internal flow, and on this basis, TRPC 6 participates in multiple signaling pathways. It can be said that TRPC6 is involved to a certain extent in the occurrence and development of tumors and plays a key role in the development of some of them. Blocking this channel can inhibit cell proliferation and block cells in the G2/M phase, while cells in the G2 phase are sensitive to radiotherapy. Therefore, blocking this calcium channel may become a target for the prevention and treatment of cervical cancer.

## Data Availability

The datasets used and/or analysed during the current study available from the corresponding author on reasonable request.

## References

[1] Monteith GR, McAndrew D, Faddy HM (2007). Calcium and cancer: targeting Ca2+ transport. Nat Rev Cancer.

[2] Pimentel AA, Benaim G (2012). Ca^2+^ and sphingolipids as modulators for apoptosis and cancer. Invest Clin..

[3] Rafiei S, Komarova SV (2013). Molecular signaling pathways mediating osteoclastogenesis induced by prostate cancer cells. BMC Cancer.

[4] Marchi S, Pinton P (2013). Mitochondrial calcium uniporter, MiRNA and cancer: Live and let die. Commun Integr Biol..

[5] Parekh AB, Putney JW (2005). Store-operated calcium channels. Physiol Rev..

[6] Ambudkar IS, Brazer SC, Liu X (2004). Plasma membrane localization of TRPC channels: role of caveolar lipid rafts. Novartis Found Symp.

[7] Bréchard S, Melchior C, Plançon S (2008). Store-operated Ca (2+) channels formed by TRPC1, TRPC6 and Orai1 and non-store-operated channels formed by TRPC3 are involved in the regulation of NADPH oxidase in HL-60 granulocytes. Cell Calcium.

[8] Ding M, Wang H, Qu C (2018). Pyrazolo[1,5-a]pyrimidine TRPC6 antagonists for the treatment of gastric cancer. Cancer Lett..

[9] Shi Y, Ding X, He ZH (2009). Critical role of TRPC6 channels in G2 phase transition and the development of human oesophageal cancer. Gut.

[10] Ding X, He Z, Shi Y (2010). Targeting TRPC6 channels in oesophageal carcinoma growth. Expert Opin Ther Targets.

[11] Zhang SS, Wen J, Yang F (2013). High expression of transient potential receptor C6 correlated with poor prognosis in patients with esophagealsquamous cell carcinoma. Med Oncol..

[12] Jose Sanchez-Collado, Jose J Lopez, Lucia Gonzalez-Gutierrez,et al. Functional role of TRPC6 and STIM2 in cytosolic and endoplasmic reticulum Ca2+ content in resting estrogen receptor-positive breast cancer cells. Biochem J. 2020;477(17):3183–3197. 10.1042/BCJ20200560 32794568

[13] Jardin I, Diez-Bello R, Falcon D (2021). Melatonin downregulates TRPC6, impairing store-operated calcium entry in triple-negative breast cancer cells. Biol Chem..

[14] Jiang HN, Zeng B, Zhang Y (2013). Involvement of TRPC channels in lung cancer cell differentiation and the correlation analysis in human non-smallcell lung cancer. PLoS ONE.

[15] Chigurupati S, Venkataraman R, Barrera D (2010). Receptor channel TRPC6 is a key mediator of Notch-driven glioblastoma growth and invasiveness. Cancer Res.

[16] Ding X, He Z, Zhou K (2010). Essential role of TRPC6 channels in G2/M phase transition and development of human glioma. J Natl Cancer Inst.

[17] Zeng B, Yuan C, Yang X (2013). TRPC channels and their splice variants are essential for promoting human ovarian cancer cell proliferation and tumorigenesis. Curr Cancer Drug Targets..

[18] Xu J, Yang Y, Xie R (2018). The NCX1/TRPC6 complex mediates TGFβ-driven migration and invasion of human hepatocellular carcinoma cells. Cancer Res.

[19] Dhennin-Duthille I, Gautier M, Faouzi M (2011). High expression of transient receptor potential channels in human breast cancer epithelial cells and tissues: correlation with pathological parameters. Cell Physiol Biochem.

[20] Wan Q, Zheng A, Liu X (2012). Expression of transient receptor potential channel 6 in cervical cancer. Onco Targets Ther..

[21] Wan Q, Chu YX, Zheng A (2011). Effects of verapamil on the proliferation and migration of cervical cancer cells. J Sichuan Univ (Med Sci).

[22] Estacion M, Li S, Sinkins WG (2004). Activation of human TRPC6 channels by receptor stimulation. J Biol Chem..

[23] Dietrich A, Gudermann T.TRPC6.HEP (2007) 179:125–141. 10.1007/978-3-540-34891-7_7 10.1007/978-3-540-34891-7_7 17217054

[24] Venkatachalam K, Montell C (2007). TRP channels. Annu Rev Biochem.

[25] Merritt JE, Armstrong WP, Benham CD (1990). SK&F 96365, a novel inhibitor of receptor-mediated calcium entry. Biochem J..

[26] El Boustany C, Bidaux G, Enfissi A (2008). Capacitative calcium entry and transient receptor potential canonical 6 expression control human hepatoma cell proliferation. Hepatology.

[27] Ge R, Tai Y, Sun Y, Zhou K (2009). Critical role of TRPC6 channels in VEGF-mediated angiogenesis. Cancer Lett.

[28] Thilo F, Liu Y, Loddenkemper C (2012). VEGF regulates TRPC6 channels in podocytes. Nephrol Dial Transplant.

[29] Madan E, Gogna R, Keppler B (2013). p53 increases intra-cellular calcium release by transcriptional regulation of calcium channel TRPC6 in GaQ3-treated cancer cells. PLoS ONE.

